# Effect of The Receptor Activator of Nuclear Factor кB and RANK Ligand on *In Vitro* Differentiation of Cord Blood CD133^+^ Hematopoietic Stem Cells to Osteoclasts

**DOI:** 10.22074/cellj.2016.4559

**Published:** 2016-08-24

**Authors:** Nasim Kalantari, Saeid Abroun, Masoud Soleimani, Saeid Kaviani, Mehdi Azad, Fatemeh Eskandari, Hossein Habibi

**Affiliations:** 1 Department of Hematology and Blood Banking, Faculty of Medical Sciences, Tarbiat Modares University, Tehran, Iran; 2 Department of Medical Laboratory Sciences, Faculty of Allied Medicine, Qazvin University of Medical Sciences, Qazvin, Iran

**Keywords:** Receptor Activator of Nuclear Factor-Kappa B, RANK Ligand, Hematopoietic Stem Cells, Osteoclasts, Calcitonin Receptor

## Abstract

**Objective:**

Receptor activator of nuclear factor-kappa B ligand (RANKL) appears to be an osteoclast-activating factor, bearing an important role in the pathogenesis of multiple myeloma. Some studies demonstrated that U-266 myeloma cell line and primary myeloma cells expressed RANK and RANKL. It had been reported that the expression of myeloid and monocytoid markers was increased by co-culturing myeloma cells with hematopoietic stem cells (HSCs). This study also attempted to show the molecular mechanism of RANK and RANKL on differentiation capability of human cord blood HSC to osteoclast, as well as expression of calcitonin receptor (CTR) on cord blood HSC surface.

**Materials and Methods:**

In this experimental study, CD133^+^ hematopoietic stem cells were isolated from umbilical cord blood and cultured in the presence of macrophage colony-stimulating factor (M-CSF) and RANKL. Osteoclast differentiation was characterized by using tartrate-resistant acid phosphatase (TRAP) staining, giemsa staining, immunophenotyping, and reverse transcription-polymerase chain reaction (RT-PCR) assay for specific genes.

**Results:**

Hematopoietic stem cells expressed RANK before and after differentiation into osteoclast. Compared to control group, flow cytometric results showed an increased expression of RANK after differentiation. Expression of *CTR* mRNA showed TRAP reaction was positive in some differentiated cells, including osteoclast cells.

**Conclusion:**

Presence of RANKL and M-CSF in bone marrow could induce HSCs differentiation into osteoclast.

## Introduction

Remodelling procedure of bone is composed of resorption and formation of this organ (1). Mesenchymal stem cells are responsible for osteoblast production during bone formation and osteoclasts are considered as bone-resorbing multinuclear cells originated from hematopoietic stem cells (HSCs) (2, 3). Physiologic condition establishes balance between osteoblast and osteoclast activities. Misbalance in the production or activity of osteoclasts causes bone diseases such as multiple myeloma (MM) (1). MM is a hematologic disease (4-7), which is determined by the monoclonal expansion of malignant plasma cells in the bone marrow (BM) (8). Bone damages including bone lesion, spinal cord compaction and also bone fracture are hallmarks of myeloma bone disease (MBD), as potential cause of myeloma patient death (9,10). Osteoclasts and osteoblasts have fundamental role in bone rebuilding and niche formation. Osteoblasts regulate osteoclast maturation and proliferation by secretion of several cytokines, such as receptor activator of nuclear factor-kappa B ligand (RANKL) and macrophage colonystimulating factor (M-CSF) (11). It has been demonstrated that circulating serum RANKL was remarkably raised in patients with MM (5). 

Recently, disturbance in osteoprotegerin (OPG)-also known as osteoclastogenesis inhibitory factor (OCIF)or tumor necrosis factor receptor superfamily member 11B (TNFRSF11B), RANKL, and RANK systems was suggested as an another mechanism for activation of osteoclasts and finally bone destruction (12). OPG is a natural soluble receptor of RANKL (6). RANKL is known as a member of the tumour necrosis factor gene family (13,14), also the main osteoclastogenic factor involved in MBD (5). M-CSF and RANKL are two important factors for Osteoclast differentiation (15,16). RANKL is expressed by osteoblasts and stromal cells of BM (17,18). Reciprocal action between RANKL and its receptor, RANK, which is expressed by osteoclasts and their precursors (19), motivate osteoclast formation and consequently bone resorption (4,5). In has been demonstrated that expression of RANK, cathepsin K (*CTSK*), tartrate-resistant acid phosphatase (*TRAP*) and calcitonin receptor (*CTR*) genes are regulated during osteoclastogenesis (20). 

Evidences show that large amount of TRAP is presented in osteoclasts. Thus, activity of this protein is considered as an established marker for identification of osteoclasts (21). 

Sadeghi et al. (22) showed that U-266 myeloma cell line and primary myeloma cells expressed *RANK* and *RANKL* mRNA. In co-culture myeloma cells with HSCs, it was also determined that expression of myeloid and monocytoid markers were increased (23). RANKL seems to be osteo clast activating factors (OAFs). 

In this study, we evaluated expression of *RANK* and *CTR* in the CD133^+^ HSCs and the differentiation capability of human cord blood hematopoietic stem cells into osteoclasts was investigated under some distinct colony-stimulating factors. 

## Materials and Methods

### Preparation of human CD133^+^ cells

In this experimental study, CD133^+^ HSCs were isolated from three samples of umbilical cord blood. A mononuclear cell fraction from cord blood was isolated by Ficoll-Paque solution (GE Healthcare Bio-sciences AB, Sweden) and centrifuged in 400g for 30 minutes at 22˚C. To remove the platelets, the cell pellet was centrifuged at 200 g for 10 minutes at 22˚C. Then, the pellet was resuspended in 500 μL of phosphate buffered saline (PBS, Medicago AB, Sweden). 50 μL of FcR blocking reagent (Miltenyi Biotec GmbH, Germany) was added, mixed well and incubated at 2-8˚C for 10 minutes. Afterwards, 50 μL of CD133 microbeads (Miltenyi Biotec GmbH, Germany) were added to the cells and incubated for 30 minutes at 4˚C. The Cells were centrifuged at 300 g for 5 minutes. The supernatant was aspirated and the cells were re-suspended in 500 μL of PBS. The cell suspension was added to a positive selection column. Column was washed with PBS. The column was removed from the magnetic separator and placed on a suitable collection tube. Enough amount of buffer was pipetted onto the column. After that, the magnetically labeled cells were flush outed by tightly pushing the plunger into the column. 

### Culture conditions for osteoclast differentiation

CD133^+^ cells were plated at a density of 7×10^4^cells/ well in 24-well plates. They were seeded in triplicate into four groups: control compared to treated groups by M-CSF, RANKL and M-CSF plus RANKL. The cells were cultured in 1mL of Iscove’s Modified Dulbecco’s Medium (IMDM, Sigma-Aldrich Chemie GmbH, Germany) containing 2 mML-glutamine (Invitrogen, CA), 100 U/mL penicillin, 100 µg/mL streptomycin (Invitrogen, CA) and 5% heat-inactivated fetal bovine serum (FBS, Invitrogen, CA). The cells in each well were separately treated by 30 ng/mL of M-CSF (R&D Systems Europe, UK), 50 ng/mL of soluble human RANKL (sRANKL, Miltenyi Biotec GmbH, Germany) and both of them. Also, cultured CD133^+^ cells in medium containing 5% FBS were used as control group. The cultures were incubated at 37˚C in a humidified atmosphere of 5% CO_2_ for 21 days. The medium was exchanged every 48 hours by demi-depletion (half of the medium was withdrawn and replenished with a fresh medium). The immunophenotyping was performed to detect the expression of CD133 and RANK within different days.

### Immunophenotyping (Flow cytometry)

For cell surface markers detection, phycoerythrin (PE)-conjugated anti-CD133 (Miltenyi Biotec GmbH, Germany) and PE-conjugated anti-RANK (Abcam Inc, USA) were used. The procedure of staining was done according to the manufacturer’s instructions. PE-conjugated mouse IgG1 isotype control antibody (Miltenyi Biotec GmbH, Germany) was used for each sample -as a negative controlto block nonspecific binding sites. After labelling, all samples were analyzed by a flow cytometer (FACSCalibur, Becton-Dickinson) in Royan Institute (Tehran, Iran). The results were analyzed by using flowjo7.6.1 software (Tree star, USA). 

### TRAP and Giemsa staining

TRAP staining of CD133^+^ cells before (day 0) and after differentiation (day 21) were done by acid phosphatase kit (Merck KGaA, Germany) according to the manufacturer’s instruction. During differentiation, CD133^+^ cells became fusiform adherent cells from day 3. These cells were detached by incubation with trypsin (Invitrogen, CA) at 37˚C for 15 minutes. Cytospins of slides were prepared and stained by TRAP and Giemsa staining. For TRAP staining, after fixation in leucognost fixing mixture, the cells were stained by freshly prepared TRAP staining solution (naphthol AS-OL phosphoric acid, sodium acetate, pararosaniline-HCL solution, nitrite solution and di-sodium tartrate). 

For Giemsa staining, cytospins of slides were immersed by putting a few drops of methanol on them and fixed in the liquid for 10 minutes. Afterwards, the slide was immersed in a freshly prepared Giemsa staining solution for 45 minutes, then rinsed with distilled water and was left to dry. Staining slides were subjected to take photo under microscope (Nikon, Japan) attached with a digital camera (SONY DSC-w7). 

### RNA preparation and polymerase chain reaction amplification

Total RNA was isolated from CD133^+^ cells before and after differentiation in control and treated groups using RNeasy Mini Kit (Qiagen, USA) based on the manufacturer’s guideline. Single strand complementary DNAs (cDNAs) were synthesized with reverse transcription (Fermentas, Denmark). Primers which were used by reverse transcriptase polymerase chain reaction (RT-PCR) technique for detection of human *RANK*, human *CTR* and hypoxanthine-guanine phosphoribosyl transferase (*HPRT1*) as internal control, were shown in the Table 1. The cDNAs were amplified using a mastercycler gradient eppendorf PCR system (Hamburg, Germany) in a mastercycler gradient eppendorf PCR system for 33 cycles. 

PCR was accomplished with the following program: 5 minutes of initial denaturation at 95˚C, 1 minute of denaturation at 94˚C, followed by the cycles comprised of annealing for 1 minute at 57˚C, extension for 2 minutes at 72˚C, terminated by a final extension incubation at 72˚C for 10 minutes. 

**Table 1:** Sequence of RT-PCR primers


Gene	primer (5ˊ-3ˊ)	Product size (bp)

* Human RANK (TNFRSF11A)*	F: TGTGGCACTGGATCAATGAG	262
	R: GTCTTGCTGACCAATGAGAG	
* Human CTR*	F: GACAACTGCTGGCTGAGTG	321
	R: GAAGCAGTAGATGGTCGCAA	
* Human HPRT1*	F: CCTGGCGTCGTGATTAGTG	125
	R: TCAGTCCTGTCCATAATTAGTCC	


RT-PCR; Reverse transcriptase-polymerase chain reaction, RANK; Receptor activator of nuclear factor-kap- pa B, TNFRSF11A; Tumor necrosis factor receptor superfamily member 11A, CTR; Calcitonin receptor, and HPRT1; Hypoxanthine-guanine phosphoribosyltransferase.

### Statistical analysis

Data were represented as the mean ± SD. Data analysis was performed using Kruskal-Wallis test. A P<0.005 was considered statistically significant. All statistical analysis was implemented with SPSS software, version 11.0 (SPSS, Chicago, IL, USA). 

### Ethical considerations

After the completion of consent form by the pregnant women, three samples of full term human umbilical cord blood were kindly provided by permission from the Iranian Blood Transfusion Organization. 

## Results

### Cell counting and viability test for CD133^+^ cells

As mentioned before, 70×10^3^ CD133^+^ cell/mL was plated in 24-well plates on the third day and divided into four groups. Within differentiation of CD133^+^ cells, these cells were counted and viability was simultaneously evaluated on days 0, 7, 14 and 21 by using methylene blue staining (Figes1, 2). 

**Fig.1:**
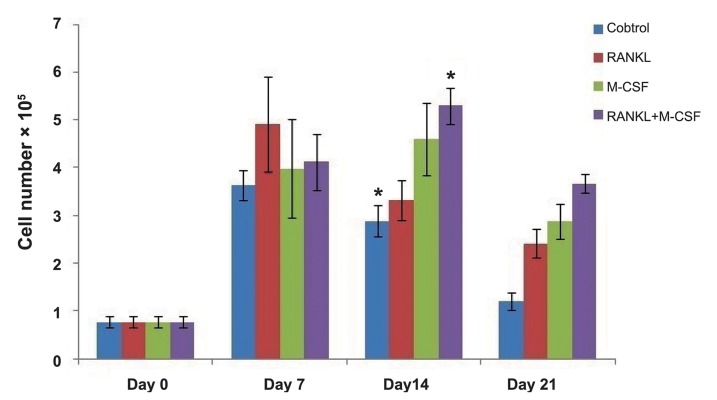
Rate of cell proliferation in the control and treated groups using various factors on days 0, 7, 14, and 21 of differentiation. These results were obtained from three replicate samples. *; Significant difference between control and treated groups by M-CSF plus RANKL in comparison with other groups (P=0.004) on day 14 of the culture, M-CSF; Macrophage colony-stimulating factor, and RANKL; Receptor activator of nuclear factor-kappa B ligand.

Using M-CSF plus RANKL, cell proliferation of control and treated groups showed a significant difference compared with the other groups (P=0.004) on day 14 of the culture (Fig.1). 

### Flow cytometric analysis of cord blood mononuclear cells

At the first day of cell isolation (before differentiation), flow cytometric results demonstrated that 93.37 and 2.74% of the cells were CD133^+^ and RANK^+,respectively.After14daysofdif-^ferentiation, RANK^+cellsincontrolaswellas^M-CSF, RANKL and M-CSF plus sRANKL treated groups were 10.63, 19.68, 18.16 and 38.80%, respectively (Fig.3). 

**Fig.2:**
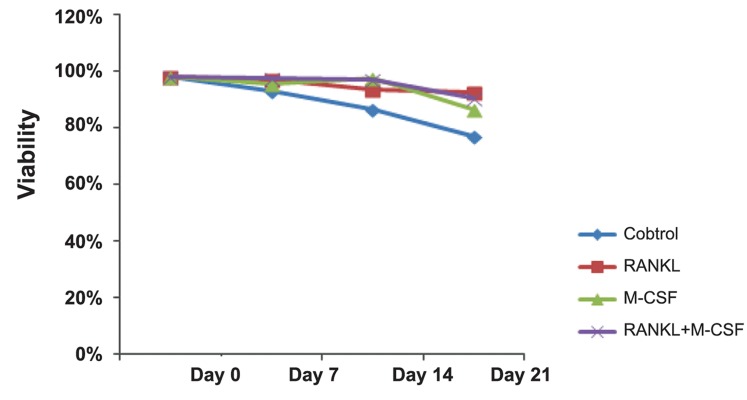
Percentage of cell viability in the control and treated groups on days 0, 7, 14, and 21 of differentiation. M-CSF; Macrophage colonystimulating factor and RANKL; Receptor activator of nuclear factor-kappa B ligand.

**Fig.3:**
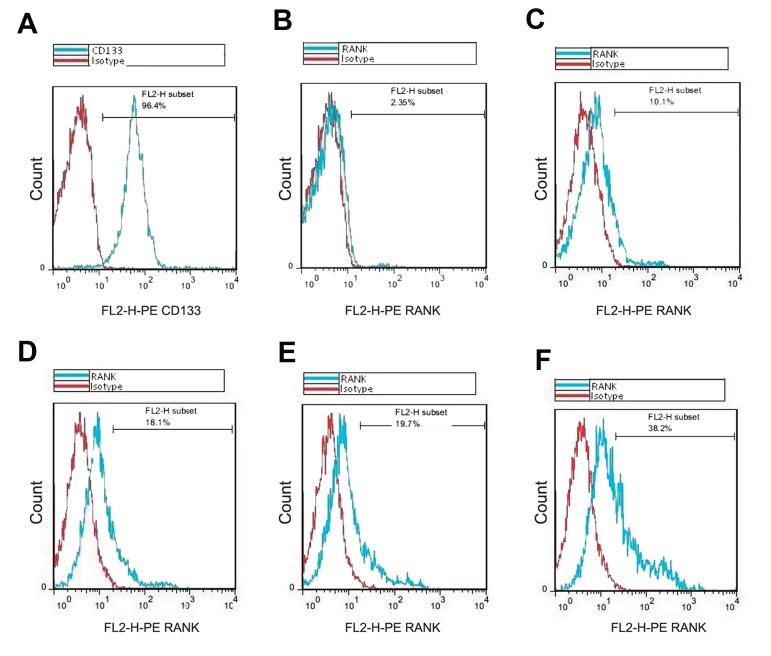
Expression analysis of surface markers CD133 and RANK using flow cytometry. A. Cord blood mononuclear cells (CBMCs) stained by PE-antiCD133, B. PE-anti-RANK antibodies on day 0 before differentiation, C. Expression of RANK marker was examined by flow cytometer, including: the control group, the cells treated by D. RANKL, E. M-CSF, and F. M-CSF plus RANKL on day 14 of differentiation. M-CSF; Macrophage colony-stimulating factor and RANKL; Receptor activator of nuclear factor-kappa B ligand.

### Gene expression 

**Fig.4:**
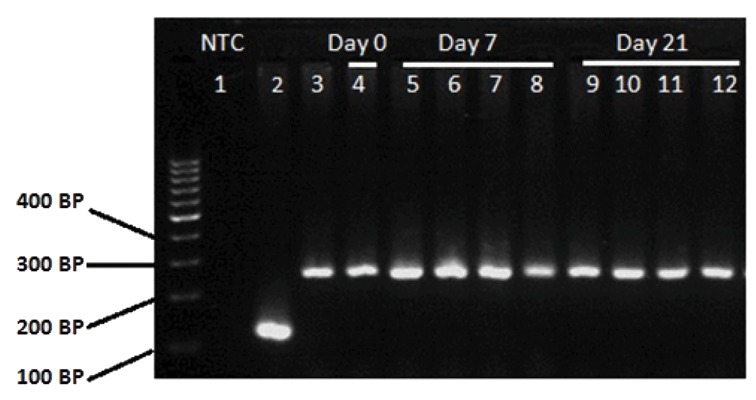
Expression of *RANK* mRNA in CD133^+^ cells demonstrated by RT-PCR. cDNA samples were obtained from: lane 1; NTC (no template control), lane 2; *HPRT1* (housekeeping gene), lane 3; Myeloma cells derived from myeloma patients (positive control), lane 4; CD133^+^ sample (before differentiation), lane 5; Control sample, lane 6; RANKL, lane 7; M-CSF, lane 8; RANKL plus M-CSF treated groups on day 7, lane 9; Control sample, lane 10; RANKL, lane 11; M-CSF, and lane 12; RANKL plus M-CSF treated groups on day 21 (after differentiation). RANKL; Receptor activator of nuclear factor-kappa B ligand, RT-PCR; Reverse transcriptase polymerase chain reaction, *HPRT1*; Hypoxan- thine-guanine phosphoribosyltransferase and M-CSF; Macrophage colony-stimulating factor.

Expression of *RANK* gene in CD133^+^ HSCs was examined by RNA extraction and RT-PCR. Before differentiation, RANK mRNA expression was detected in CD133^+^ HSCs. Results showed that cultured CD133^+^ cells on days 7 and 21 of the differentiation expressed RANK at mRNA level in control and each of RANKL, M-CSF and RANKL plus M-CSF treated groups (Fig.4). 

**Fig.5:**
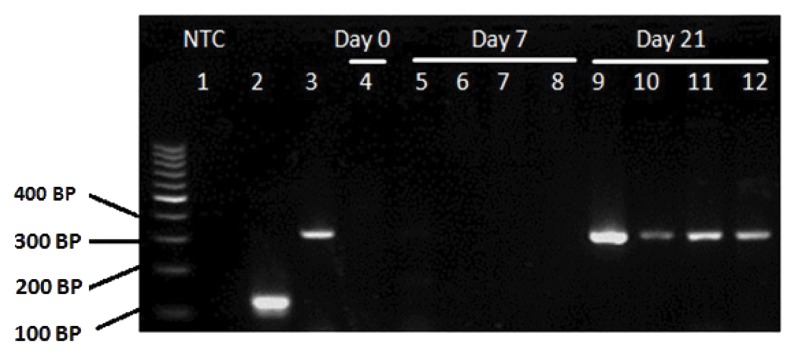
Expression of *CTR* mRNA in CD133^+^ cells, demonstrated by RT-PCR. cDNA samples were obtained from: lane 1; NTC (no template control), lane 2; HPRT1 (housekeeping gene), lane 3; *MCF-7* (positive control), lane 4; CD133^+^ sample (before differentiation), lane 5; Control group, lane 6; RANKL, lane 7; M-CSF, lane 8; RANKL plus M-CSF treated groups on day 7, lane 9; Control group, lane 10; RANKL, lane 11; M-CSF, and lane 12; RANKL plus M-CSF treated groups on day 21 (after differentiation). *CTR*; Calcitonin receptor, RANKL; Recep- tor activator of nuclear factor-kappa B ligand, RT-PCR; Reverse transcriptase-polymerase chain reaction, HPRT1; Hypoxanthine-guanine phosphoribosyltransferase, and M-CSF; Macrophage colony-stimulating factor

RT-PCR results demonstrated that CD133^+^ cells on the first day of isolation (before differentiation) do not express *CTR* gene at mRNA level, however it was detected after 21 days of differentiation in control and treated groups by RANKL, M-CSF and RANKL plus M-CSF (Fig.5). 

### TRAP staining

**Fig.6:**
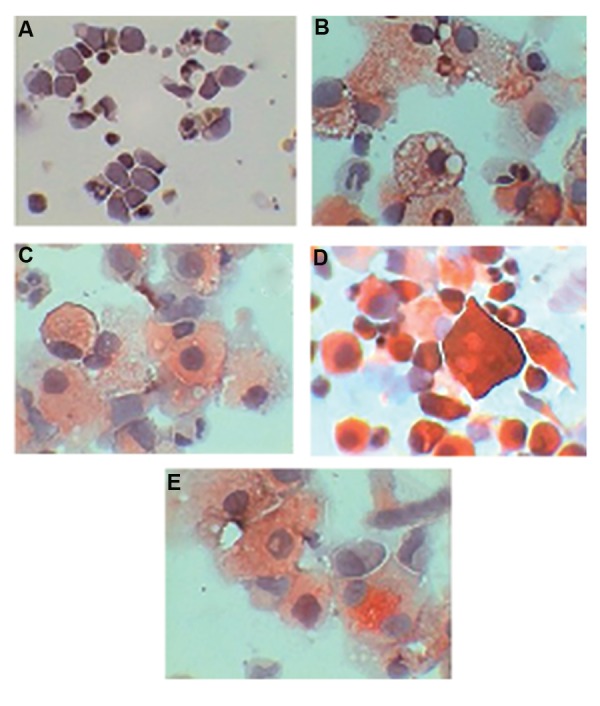
Results of TRAP staining in CD133^+^ cells, A. Before differentiation and after 21 days of differentiation by B. M-CSF, C. Control group, D. RANKL plus M-CSF, and E. RANKL. TRAP; Tartrate-resistant acid phosphatase, M-CSF; Macrophage colony-stimulating factor and RANKL; Receptor activator of nuclear factor-kappa B ligand.

The results showed that CD133^+^ cells were all negative for TRAP staining on the first day of isolation. As illustrated in Figure 6, treated cells with M-CSF showed TRAP-positive by clear vacuoles in the cytoplasm. Cells treated by RANKL plus M-CSF was determined as a small number of large multinucleate cells with strong TRAP staining representation (Fig.6). The reaction of TRAP was partially positive in the control and treated group by RANKL.

### Giemsa staining

**Fig.7:**
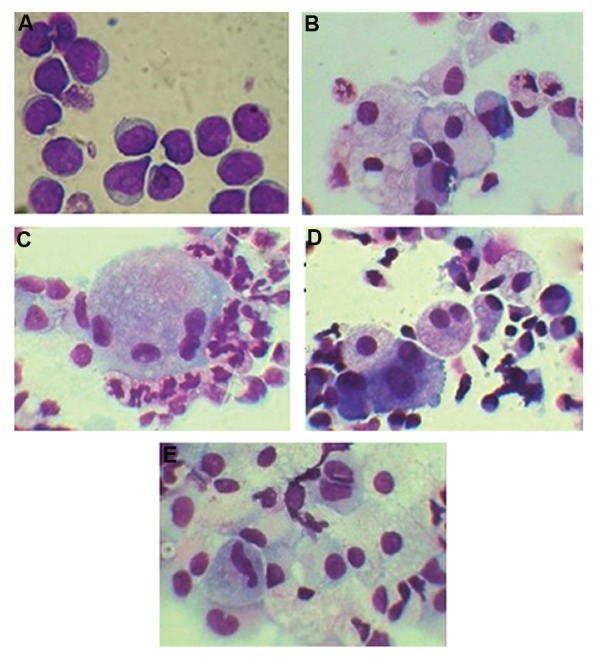
Results of Giemsa staining in CD133^+^ cells, A. Before differentiation and after 21 days of differentiation by: B. M-CSF, C. RANKL plus M-CSF, D. RANKL, and E. Control group. M-CSF; Macrophage colony-stimulating factor, and RANKL; Receptor activator of nuclear factorkappa B ligand.

Following preparation of smears by cytospin, morphology of CD133^+^ cells before and after differentiation was examined using Giemsa staining. Before differentiation, cells were immature showing a large nuclear to cytoplasmic ratio, condensed chromatin and scanty cytoplasm (Fig.7). However, treated cells by using any of RANKL, M-CSF, RANKL plus M-CSF or control cells were mature and binuclear after differentiation. Treated cells by M-CSF were identified as vacuolated macrophages. Also, multinucleated cells (≥3 nuclei) were seen in addition to the binuclear cells in treated group by RANKL plus M-CSF (Fig.7).

## Discussion

Sadeghi et al. (22) showed that human U-266 cell line and primary myeloma cells expressed *RANK* and *RANKL* at mRNA levels and it can cause myeloma cell auto activation. Also, our findings demonstrated increment in expression of myeloid and monocytoid markers in co-culturing of myeloma cells with HSCs (23). We indicated that the co-culturing of myeloma cells with cord blood HSCs cause to HSCs differentiation to osteoclastic lineage and it may happened in BM. A number of potentially OAF markers, like IL-1β, IL-6, TNF-α were detected in MM. However, none of these markers were available at high level in the majority of the respective patients. So, these markers cannot be considered as primary inducers of osteoclast activation in MM (10). CBMC are known as a superior source of human osteoclast precursors (24). Many studies have demonstrated formation of human osteoclast using adherent or CD14 positive fraction of PBMCs or granulocyte/ macrophage colony forming units (CFU-GM) obtained from the BM (25). The expression of *RANK* and *CTR* has not been reported in human umbilical cord blood HSCs, yet. Therefore, this investigation covered two aspects. First, we examined the expression of *RANK* and *CTR*. Second, the differentiation capability of human cord blood hematopoietic stem cells to osteoclast was investigated. 

In the present study, for the osteoclast differentiation of CD133^+^ HSCs, M-CSF and a soluble form of RANKL were used instead of stromal cells. 

M-CSF is an essential factor which is produced by stromal cells (13). Survival, proliferation, and *RANK* expression are induced in mononuclear phagocyte precursors by M-CSF (26). 

In this study, after isolation, flow cytometric analysis of CD133^+^ cells on day 0 showed that 2.7% of the respective cells are RANK^+^. According to Arai and colleagues, fluorescence analysis cell sorting (FACS) showed that 5.4% of the mouse BM cells are RANK^+^(13). RT-PCR results suggested that human CD133^+^ cord blood cells express RANK at mRNA level on day 0, before differentiation. How- ever, *CTR* mRNA was not detected at similar day. We also determined that CD133^+^ HSCs express *RANK* on days 7 and 21 of differentiation, at mRNA level. Ciraci et al. (27) showed that MUTZ-3-CD34^+^ cells induced by M-CSF, RANKL and TNF-α expressed *RANK* mRNA from day 3 of culture. Lari et al. (28) showed that human peripheral blood monocytes expressed RANK mRNA on day 14, while they were treated by M-CSF and RANKL

In this work, RT-PCR results showed that by day 7 of differentiation, CBMCs were not able to express *CTR* mRNA. However, it could be expressed on day 21 of differentiation. It is proposed that expression of *CTR* mRNA is associated with increased length of incubation. CTR is considered as main marker of osteoclast establishment and maturation (26). Kurihara et al. (29) showed that mononuclear precursors of human marrow expressed CTR.

We also found that CD133^+^ cells could express *CTR* mRNA when cultured in IMDM+5% FBS. Here, flow cytometric results on day 14 showed that the percentage of RANK^+^ cells increased with longer incubations in the presence of RANKL plus M-CSF compared to the case treated by individual RANKL or M-CSF (2.7% for day 0 and 38.8% for days 14).

Staining results indicated TRAP-negative cells before differentiation of CD133^+^ cells, on day 0, and TRAP-positive cells after differentiation on day 21. It is suggested that variation of cytoplasmic TRAP staining in the control and the treated groups by RANKL or M-CSF could highlight different stages of cell maturity. TRAP is known to be an inducible enzyme, whose expression is related to cell growth and differentiation (30).

Miyamato and colleagues determined when osteoclast precursor cells derived from mouse BM were cultured in the presence of M-CSF plus RANKL or M-CSF, they generated TRAP^+^ cells (31). On the other hand, TRAP^+^ cells were not determined when these cells were only treated by M-CSF (13).

As previously indicated, in addition to the presence of TRAP^+^ cells in the treated groups, we determined the respective cells in the control group. Faust and colleagues showed that cultured human PBMC in aMEM+10% FBS without the addition of stromal cells, growth factors or cytokines led to TRAP^+^ cells with low levels of TRAP expression (30).

## Conclusion

Presence of RANKL and M-CSF in bone marrow could induce osteoclast differentiation from HSCs. Our findings indicated that RANK and RANKL lead to the deregulation of bone remodelling, increment of osteoclast activity and bone destruction in myeloma patients with bone disease. 

Therefore, we propose to determine the boneresorbing capacity of the differentiated cells by pit formation assay for monitoring novel drugs. If these cells are capable to create bone resorption cavity, we can use cord blood sample to generate osteoclasts *in vitro* instead of using invasive technique to obtain BM. 
